# Retinal Responses to Short- and Longer-Term Predominant ON or OFF Stimulation in Emmetropes and Myopes

**DOI:** 10.1167/iovs.66.2.66

**Published:** 2025-02-26

**Authors:** Sandra Wagner, Annemarie Settele, Torsten Strasser

**Affiliations:** 1 Institute for Ophthalmic Research, University of Tuebingen, Tuebingen, Germany; 2 Royal Institute of Technology (KTH) – AlbaNova, Stockholm, Sweden; 3 Herbert Wertheim School of Optometry and Vision Science, University of California, Berkeley, California, United States; 4 Eye Hospital Tuebingen, Tuebingen, Germany

**Keywords:** myopia, electroretinography (ERG), ON/OFF pathways, nearwork, adaptation

## Abstract

**Purpose:**

The link between nearwork and myopia is controversially discussed. Features of the viewing target may stimulate eye growth, for example, black-on-white text was found to stimulate retinal OFF pathways and promote choroidal thinning, whereas inverted text led to ON pathway stimulation and thicker choroids. We used electroretinograms (ERGs) to compare retinal activity for both stimuli in the parafovea in emmetropes and myopes and studied the effects of adaptation.

**Methods:**

ERGs were recorded in 42 subjects (18–30 years) during 200 ms-flashes on a CRT monitor, superimposed with an annulus or circles filled with gray or inverted or standard text. Ganzfeld ERGs (500 ms) were taken before and after 30 minutes of reading standard or inverted text at 25 cm to determine adaptation effects. The ON- (b-wave) and OFF-responses (d-wave) were analyzed using linear mixed effects models and pointwise *t*-testing.

**Results:**

(1) Stimulus size affected retinal ON-responses of both groups (*p* < 0.001), with larger responses to a 6 to 12 degrees annulus than to a 12-degree circle. (2) Myopes displayed larger ON-responses to inverted text contrast than emmetropes within 6 to 12 degrees. (3) After adaptation to text, ON-responses were reduced (*p* = 0.010) irrespective of refraction and contrast. (4) Emmetropes showed reduced ON- and OFF-responses to inverted text contrast. (5) Only emmetropes had reduced ON- and larger OFF-responses after adapting to standard text.

**Conclusions:**

Myopes had largest ON-responses with inverted contrast in the perifovea. Emmetropes displayed larger adaptive changes after ON/OFF stimulation. In both groups, inverted contrast still reduced ON-responses, suggesting that efficient activation of retinal ON channels to inhibit myopia might require additional OFF channel suppression.

Myopia is not merely a refractive error correctable with spectacles or contact lenses. The excessively elongated eye increases the risk of severe ocular diseases and might thus turn myopia into a significant health problem and burden for the affected child.^1^ With worldwide increasing numbers of myopic children, governments and public health systems urgently need improved means for myopia prevention and control, as also reflected in the World Health Organization's latest report.^2^ Although genetics undoubtedly has an impact on the odds of becoming myopic,^3^ the rapid increase in myopia prevalence is only explained by additional environmental risk factors.^4^ The suggestion that myopia development might be linked to nearwork dates back to the time of Kepler.^5^ Research over the last decades provided evidence for this hypothesis by revealing an association between myopia prevalence and years of education and the amount of close work being performed.^6^ Significant differences between myopic and non-myopic eyes as for their accommodation response behavior,^7^ their ciliary muscle structure,^8^^,^^9^ and its activity during accommodation^10^ were also found.

However, nearwork as a possible environmental risk factor is still being controversially discussed.^11^ The mechanism of how nearwork and especially reading promotes the eye's elongation has remained unclear. Suggestions that the lack of higher spatial frequencies indoors,^12^ the defocus experience,^13^ or the body posture during close work^14^ are involved, have been put forward. Recent studies pointed to a possible relationship with the retinal ON and OFF pathways. Animal models, using pharmacological^15^ or genetic methods,^16^ had already shown that the selective inhibition or blocking of ON/OFF pathways affects the refractive development, suggesting an association between myopia and reduced activation of the ON channel. Subsequently, studies in humans showed that the retinal ON and OFF channels are selectively activated depending on the specific contrast polarity of the text: black-on-white text led to an enhanced stimulation of the retinal OFF pathway, but inverted contrast to a predominant ON activation. In turn, the thickness of the choroid, the highly perfused layer nourishing the retina, increased with ON and decreased with OFF stimulation.^17^ Studies in animal models provided evidence that choroidal thinning precedes myopia development, whereas a thickening is associated with slower eye growth.^18^^,^^19^ Moreover, ON bipolar cells interact with dopaminergic amacrine cells.^20^ The predominant stimulation of the ON pathway might increase retinal dopamine levels, which inhibits eye growth in animal models.^21^ In mice, form deprivation myopia was inhibited by bright light stimulation, leading to increased activity of dopamine receptors of the ON pathway.^22^ The chicken model revealed intermittent bright light to be more potent than continuous exposure.^23^ The positive effects of bright light exposure for protecting from myopia onset also become apparent from recent studies in children.^24^^–^^26^

During accommodation, axial length of the eye increases^27^ because the choroid becomes thinner.^28^ This thinning was found to be further strengthened when using a standard black-on-white text during the reading task, thereby mainly stimulating the retinal OFF pathway.^29^ A further study revealed that reading of standard contrast text as compared to the situation during walking leads to a reduced spatiotemporal contrast. The luminance distribution was found to be biased toward dark contrasts in the fovea. Both could potentially contribute to a reduced visual ON pathway stimulation.^30^ In a previous study using pattern electroretinography (ERG), we found significant differences in retinal responses to standard or inverted contrast between emmetropes and myopes, which additionally depended on the retinal area of stimulation.^31^ A study with young adults found that the selective activation of retinal pathways over a 28-minute period using sawtooth luminance profiles induced significantly improved visual acuity with ON stimulation in bright and OFF stimulation in dark conditions.^32^ Contradicting findings of the strength of selective retinal pathway activation were previously also reported: chicks that were reared under dynamic ON stimulation with flickering light over a 1-week period while wearing negative lenses showed increased retinal dopamine release. But the animals developed more myopia than the control group reared under room light.^33^ Also, young adults who read inverted contrast text that was additionally spatially filtered to simulate myopic longitudinal chromatic aberration had longer eyes after 30 minutes of reading.^34^ Moreover, a prolonged selective ON stimulation might induce adaptation effects which could counteract the aim of inhibiting eye growth. It is also still unknown how a longer period of selectively stimulating the retinal pathways might affect the neural responses of the retina in human eyes of different refractive errors. In this study, we aimed to assess how 30 minutes of reading text of either standard or inverted contrast at close distance affects the retinal processing of myopic as compared to emmetropic eyes. Additionally, we tested whether effects of selective ON and OFF stimulation on retinal activity can be measured immediately without any adaptation. The outcome of our exploratory investigation provides new information about the retinal ON/OFF system in myopia. This might lead to recommendations for improved nearwork behavior in children at school or during leisure time to possibly support myopia control therapy.

## Methods

**Figure 1.**
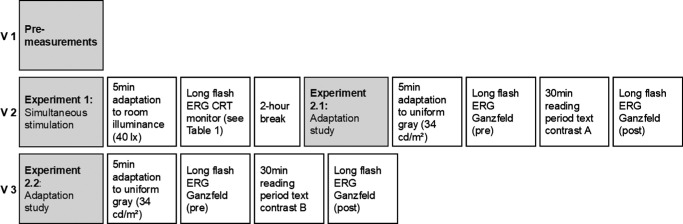
Experimental procedure. Description of the three study visits.

The study was approved by the Ethics Committee of the Medical Faculty of the University of Tuebingen, Germany (155/2022BO2) and performed in line with the Declaration of Helsinki. Young adults between 18 and 30 years of age with either emmetropic or myopic refractive state (spherical equivalent refractive error [SER] between –6.0 diopter [D] and +0.5 D) were recruited from the students of the University of Tuebingen, Germany. They were informed about the study's procedures, data processing, and their rights when participating in the trial. Signed consent about the procedures, data processing, and their right to withdraw from the study without explanation was obtained. During the first visit, general and ocular health history were recorded and an ophthalmological assessment to confirm ocular health (anterior segment and fundus biomicroscopy) as well as pre-measurements were performed. These included objective refraction (AR-360A; Nidek Co., Ltd, Gamagori, Aichi, Japan), subjective refraction with measurement of monocular and binocular best-corrected distance visual acuity, and intraocular pressure (IOP) measurement (iCare IC-100, Vantaa, Finland). Additionally, maximal subjective accommodation amplitude was determined by the push-up method. If in agreement with the inclusion criteria, the volunteers were enrolled in the trial and a second appointment was arranged. During the second visit, experiment 1 as well as the first part of experiment 2 were performed. The second part of experiment 2 was undertaken on a third visit (see Fig. 1). During the entire study, subjects with myopia had their sight corrected with soft contact lenses. Electroretinographic measurements from the right eyes were taken under light-adapted conditions using a visual electrophysiology recording system (Espion e²; Diagnosys LLC, Cambridge, UK). After having cleaned the respective skin areas to improve conductivity, the ground electrode was placed on the forehead, the counter electrode on the temple, and a Dawson, Trick, and Litzkow (DTL) electrode^35^ was placed along the right eye's lower eyelid.

### Experiment 1: Simultaneous Stimulation

In the first measurement, a long flash ERG was taken while stimulating either the retinal ON or OFF pathway independently. The experimental procedure was similar to our previous study.^31^ The subjects were exposed to 10 different stimuli presented on a CRT monitor (Diamond Plus 230SB, Mitsubishi) at 40 cm viewing distance while long flash ERGs were sequentially recorded. The stimuli were generated using PsychoPy^36^ and synchronized by an external trigger: a 200 ms-flash (120 cd/m² on 30 cd/m² background; 60 repeats) was overlaid by (i) an annular area, centered around the fovea between 6 degrees and 12 degrees, (ii) a circular area of 12 degrees, and (iii) a circular area of 6 degrees, each consecutively filled with (1) uniform gray, (2) inverse contrast text, and (3) standard contrast text, being isoluminant (53 cd/m²), lastly followed by a 200 ms-flash only (Supplementary Fig. S1, see the Table). The text (letter height 0.57 degrees, line spacing 0.9 degrees, and font style Open Sans) within the patterns changed continuously after 16 frames. Room illuminance was set to approximately 40 lx.

**Table.** Order of Stimulus Presentation in Experiment 1

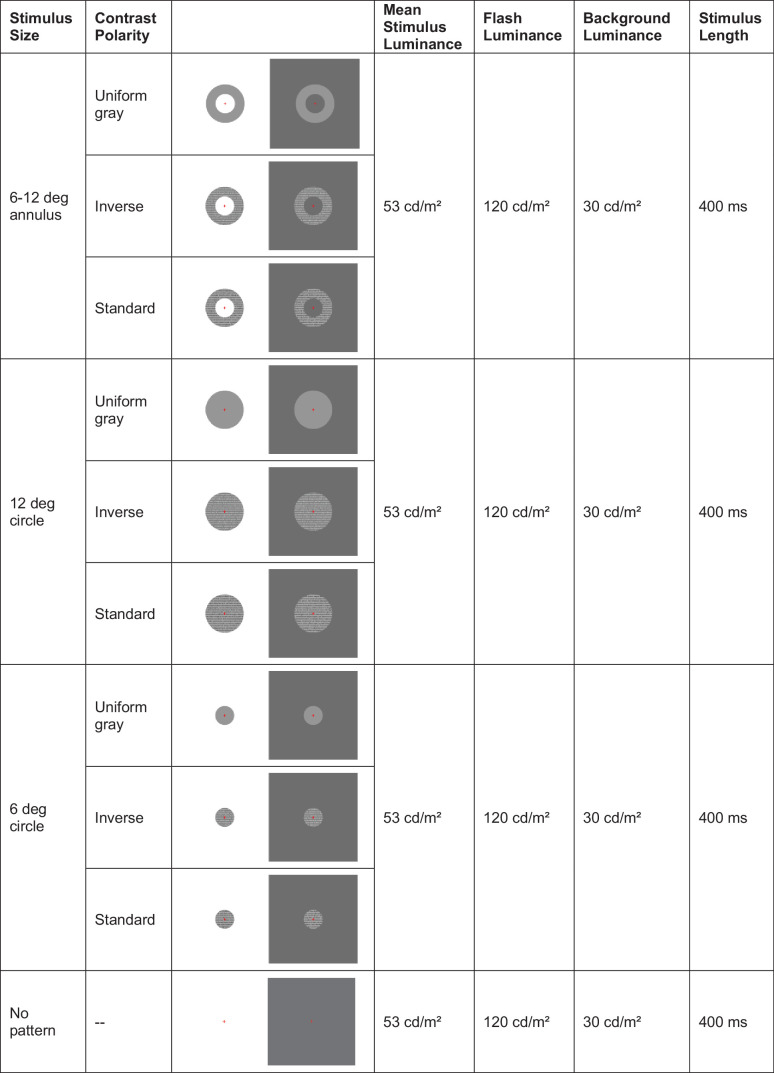

Long flashes were superimposed by stimuli of different sizes and fillings (see also Supplementary Fig. S1).

### Experiment 2: Adaptation Study

**Figure 2.**
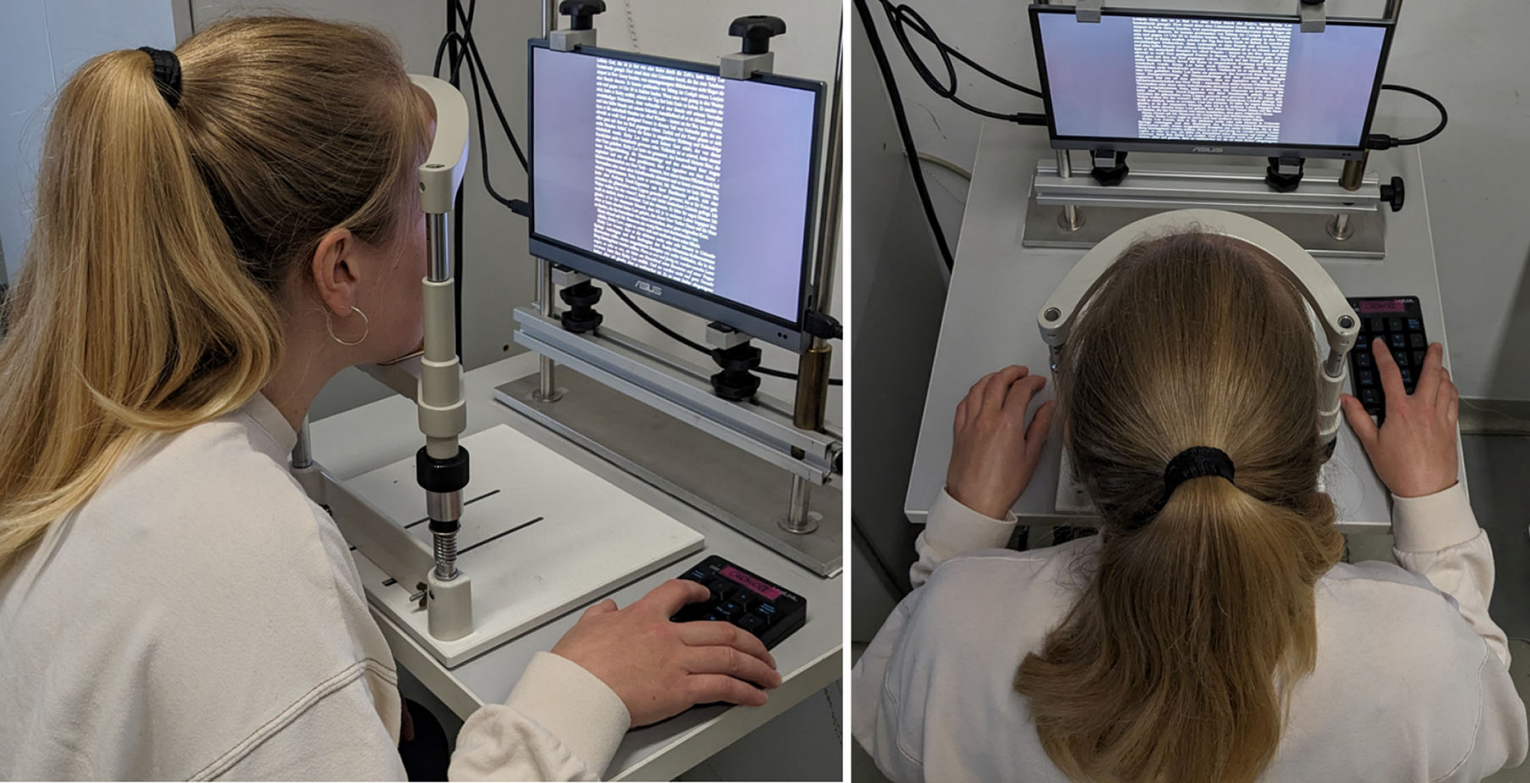
Subject position during 30-minute reading task of experiment 2 (here, OFF stimulation using black-on-white text).

In the subsequent adaptation study, the selective ON or OFF stimulation was achieved by presenting a novel as a continuous text with a visual angle of 0.57 degrees (Times New Roman, font size 13 pixels, maximum letter height 2.5 mm) on a display placed at 25 cm (13.3 inch OLED monitor, ASUS; resolution 1920 × 1080) either with bright letters on dark background (ON) or dark letters on brighter background (OFF). Both conditions were matched in luminance (34 cd/m²) with a Michelson contrast of 86%. Subjects were provided with a keypad to scroll through the text themselves (Fig. 2). The presentation order for the choice of text contrast polarity was randomized, and the respective measurements were performed at separate study visits. To adapt to the mean luminance of the following task (34 cd/m²), the subjects were placed in front of the reading device, using a head rest at 25 cm distance, and asked to look at the gray display for a period of 5 minutes. This was followed by the baseline ERG measurements, which were taken from the right eyes while presenting full-field stimuli (ColorDome, Espion e²; Diagnosys LLC, Cambridge, UK). A light-adapted On-Off ERG (500 ms length, 30 cd/m² vs. 330 cd/m²) was measured according to recommendations by the International Society for Clinical Electrophysiology of Vision (ISCEV)^37^ with 2 repetitions of 20 sweeps. Subsequently, the subjects were asked to perform the reading task for 30 minutes. Immediately after the task completion, ERG recordings were repeated using the same protocol as before. After the last recording, the subjects’ eyes were reassessed for ocular health by an ophthalmologist using the slit lamp, and IOP was again measured. At the third visit (see Fig. 1), ERG recordings were repeated using the same protocol but applying the opposite contrast polarity for the reading task.

### Statistical Analysis

ERG data were filtered with a bandpass (0.312 Hertz [Hz] to 300 Hz), followed by a manual filtering of the individual traces for blink artifacts. Data analysis was performed using JMP 16 (SAS Institute GmbH, Heidelberg, Germany).

To statistically assess experiment 1, the simultaneous ERG recordings on the CRT monitor, a linear mixed-effects model (LMM) was used with amplitudes and implicit times of b- and d-wave as dependent variables and the independent variables refraction (emmetropic/myopic), size (6 degrees, 12 degrees, and 6–12 degrees), contrast polarity (inverted, standard, and gray), their interactions, and subject as a random variable. Tukey’s honestly significant difference (HSD) test was used for post hoc analysis. To allow an evaluation of the retinal response changes over time, we applied pointwise t-testing and used the 95% confidence intervals (CIs) to compare the refractive groups (difference of the means) and the different pattern fillings (mean differences). As described above, the custom-made stimuli were presented on a CRT monitor. In contrast to recordings obtained with the integrated Ganzfeld device of the Espion system, this setup did not allow for recording a pre-stimulus interval for baseline removal. Furthermore, the elicited ERG responses were generally smaller and more prone to artifacts. To process the signals, each individual trace was therefore detrended by subtracting a fitted third-order polynomial (equivalent to high-pass filtering), followed by symmetric smoothing with a 10-ms window (equivalent to low-pass filtering) to reduce high-frequency noise. An additional baseline removal was omitted to maintain data integrity. Finally, individual traces were averaged per subject and condition, and a pointwise *t*-test was used to assess differences between conditions and refractive groups. Retinal response changes of b-, and d-waves, as well as of the area of the photopic negative responses (PhNR) were evaluated. The latter arise in response to a flash as a negative potential after the b-wave (PhNR_on_), and in case of longer flashes also after the d-wave (PhNR_off_). The PhNR represent the general activity of retinal ganglion cells and were found to be reduced in retinal diseases.^38^^,^^39^

As for experiment 2, the adaptation study, a LMM served to assess effects of the independent variables refraction (emmetropic/myopic), measurement time (pre/post), condition (ON stimulation/OFF stimulation), and their interactions on the dependent variables amplitude and implicit time of b- and d-wave of the On-Off ERG. Having performed two repetitions of the ERG for each subject and contrast condition, respectively, we added the result number (1/2) as a factor to test the ERG repeatability. The subject was added as a random variable to account for intersubject variability and the repeated measurements. Again, pointwise t-testing was used to assess retinal responses over time. Therefore, a baseline correction of the OFF-response of the On-Off recording was undertaken by subtracting the mean value of the first 10 ms from the single recordings per subject and condition. Subsequently, the average of the 2 repetitions and the 95% CIs of the mean differences (pre/post; contrast) or differences of the means (groups) were calculated and compared between refractive groups and conditions.

## Results

ERGs were recorded from 21 emmetropic (7 male subjects, age = 25.29 ± 2.37 years, SER OD = –0.07 ± 0.22 D) and 21 myopic (8 male subjects, age = 25.10 ± 3.19 years, SER OD = –2.49 ± 1.06 D) young adults. The measured right eye's maximum accommodation amplitude matched expectations from their ages with 8.42 ± 2.00 D in the emmetropic and 9.00 ± 2.02 D in the myopic group, respectively.

### Simultaneous Selective ON/OFF Stimulation

Regarding the simultaneous ERG recordings, the results of 2 subjects were excluded due to an increased number of artifacts arising from high blink rates, leaving a dataset of 20 emmetropes (6 male subjects, age = 25.35 ± 2.41 years, SER OD = –0.07 ± 0.23 D) and 20 myopes (7 male subjects, age = 25.05 ± 3.27 years, SER OD = –2.46 ± 1.08 D).

#### Analysis of ERG Amplitudes and Implicit Times

The LMM revealed a statistically significant effect of the stimulus size on the b-wave amplitudes (F_2,304_ = 24.89, *p* < 0.001), irrespective of the contrast polarity or refractive error. The post hoc test showed that the ON-response for the 6 degrees circle was significantly larger than for the 12 degrees circle (Δ_6 deg-12 deg_ = 1.37 µV, 95% CI = 0.91 to 1.82) and the 6–12 degrees annulus (Δ_6 deg-(6-12 deg)_ = 0.78 µV, 95% CI = 0.32 to 1.24; both *p* < 0.001). Additionally, the ON-response for the annulus was significantly larger than for the 12 degrees circle (Δ_(6–12 deg)-12deg_ = 0.58 µV, 95% CI = 0.13 to 1.04, *p* = 0.008). The implicit times of the OFF-response were dependent on the polarity (F_2,304_ = 4.07, *p* = 0.018), with significantly longer times for a filling with gray than with inverted contrast text (Δ_gray-inverted_ = 1.04 ms, 95% CI = 0.10 to 1.98, *p* = 0.026).

#### Comparison Between Retinal Responses of the Two Refractive Groups

**Figure 3.**
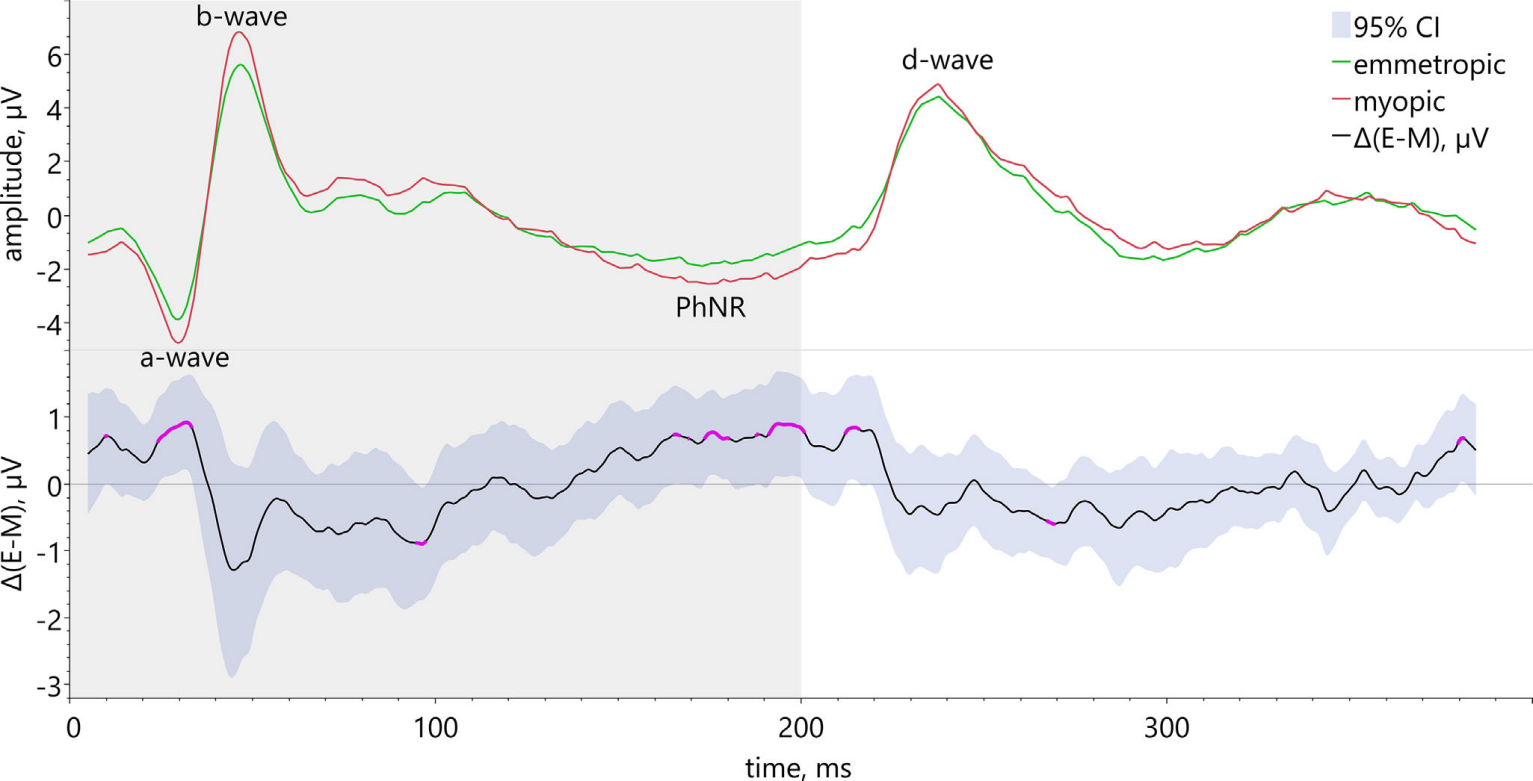
Comparison between mean On-Off ERG responses of emmetropic (*green line*, *n* = 20) and myopic eyes (*red line*, *n* = 20) with lower graph (*black line*) depicting the mean difference Δ(emmetropic-myopic) with the 95% confidence intervals and significant results highlighted (*pink*). Labels define ERG a-, b-, and d-wave as well as the area of PhNR_on_.

The analysis of the 95% CIs with pointwise t-testing showed for the On-Off ERG without any additional stimulus statistically significant differences between emmetropic and myopic eyes. Myopic subjects had larger amplitudes in the time ranges attributed to the a-wave and the PhNR, whereby the area within 170 ms to 190 ms is assumed to correspond to the PhNR_on_ (Fig. 3).

**Figure 4.**
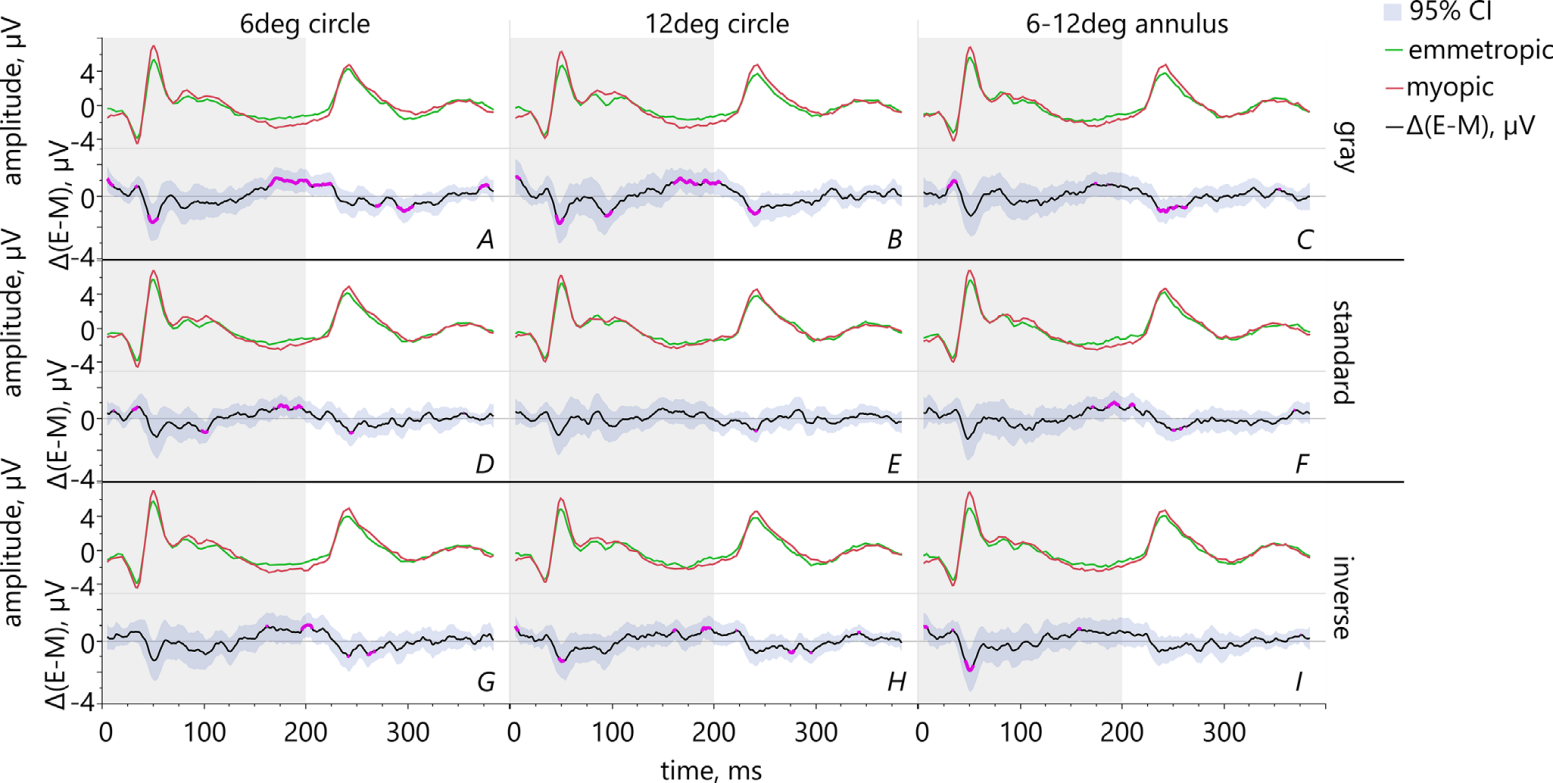
Comparison between ERG responses of emmetropic (*green line*) versus myopic (*red line*) participants for different sizes and polarity of stimuli using pointwise t-testing. The lower graphs (*black line*) show the mean difference between the two refractive groups and 95% confidence intervals. The pink areas highlight statistically significant differences.

Statistically significantly larger PhNR in myopic eyes compared to emmetropic eyes were confirmed for the 6 degrees circle filled with gray (Fig. 4^5^,^6^,^7^A) and with standard contrast (Fig. 4D). The same holds true for the 12 degrees circle gray (Fig. 4B) and the annulus stimulus filled with standard contrast text (Fig. 4F). The ON-responses of myopes were significantly larger than those of emmetropes for 6 degrees and 12 degrees circles when filled with gray (see Figs. 4A, 4B). This was also the case for the conditions 12 degrees and 6–12 degrees filled with inverted contrast text (Figs. 4H, 4I). Furthermore, myopes revealed significantly larger OFF-responses for the condition gray with stimuli sizes 12 degrees and 6–12 degrees as compared to emmetropic eyes (see Figs. 4B, 4C).

**Figure 5.**
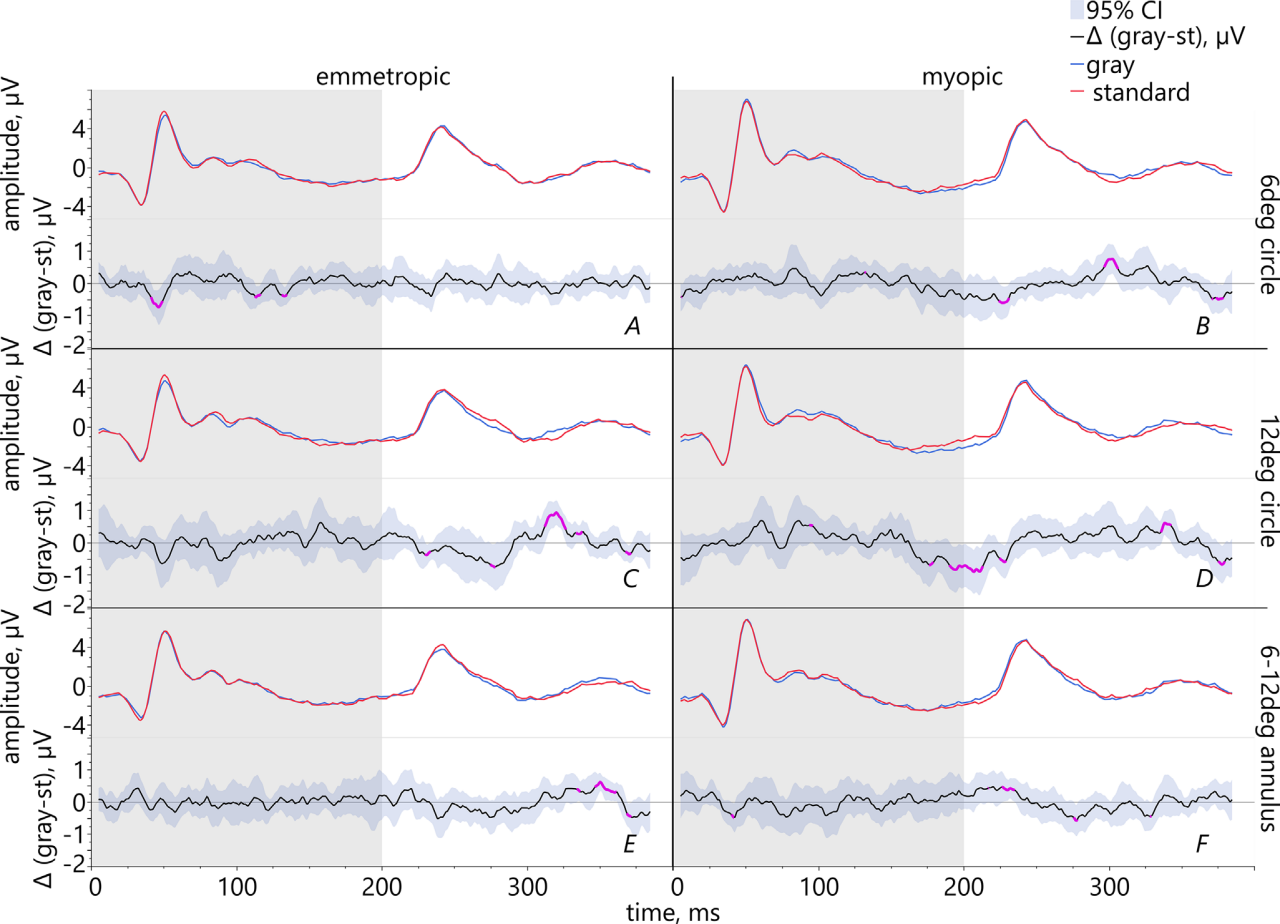
Pairwise comparison between the conditions of the pattern filling gray (represented by the *blue lines*) and standard contrast (*red lines*) in emmetropic (*left*) and myopic group (*right*). Lower graphs (*black line*) show mean difference with 95% confidence intervals and significant differences (highlighted in *pink*).

**Figure 6.**
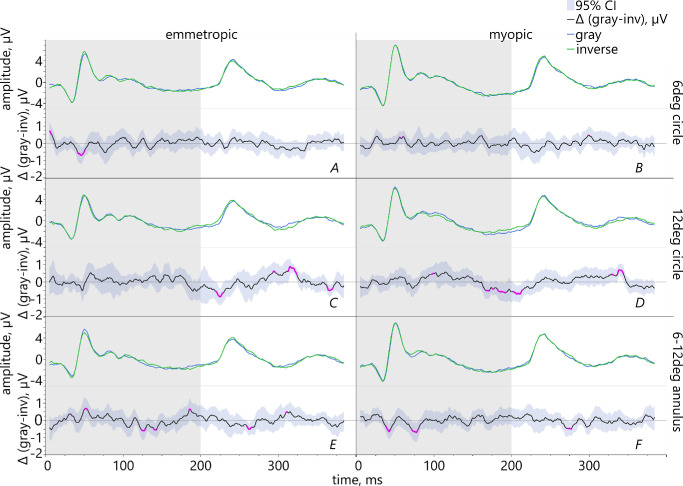
Pairwise comparison between the conditions gray (represented by *blue line*) and inverted contrast text (represented by the *green line*) with the mean differences (*black line*) and 95% confidence intervals in the lower graphs. The pink areas highlight the significant results.

**Figure 7.**
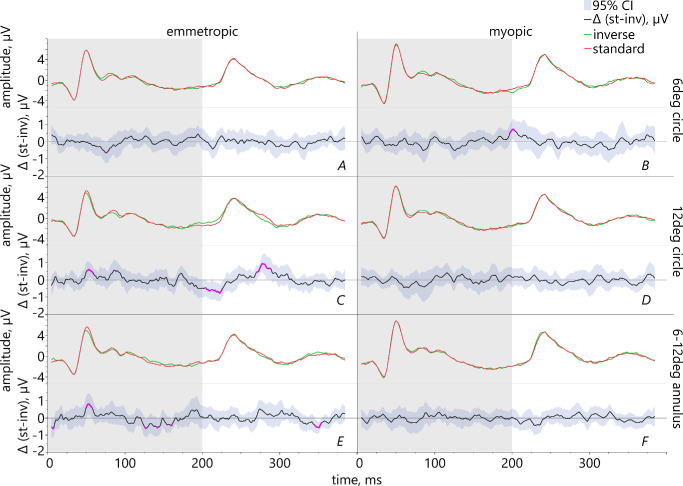
Pairwise comparison between the conditions inverted (shown as the *green line*) and standard contrast text (*red line*) in the two refractive groups and the respective mean differences between these conditions (*black line*) with 95% confidence intervals and significant outcomes (highlighted in *pink*).

#### Comparison Between Retinal Responses to Different Pattern Fillings

The pairwise comparisons of the three pattern fillings (i.e. gray versus standard contrast text, gray versus inverted contrast text, and standard versus inverted contrast text) were performed separately for both refractive groups:

(1) Comparing the fillings gray versus standard contrast text showed that emmetropes had significantly larger ON-responses for a 6 degrees stimulus which was filled with standard contrast than with gray (Fig. 5A). Myopic retinae exhibited significantly larger PhNR for a 12 degrees circle filled with gray than with standard contrast (Fig. 5D).
(2) The comparison gray versus inverted contrast showed that in emmetropes, ON-responses were significantly increased for inverted contrast within a 6 degrees area (Fig. 6A), but reduced in the area 6–12 degrees (Fig. 6E). Myopes had significantly larger PhNR for the 12 degrees stimulus filled with gray than with inverted contrast text (Fig. 6D).
(3) Comparing the conditions standard versus inverted contrast text revealed significantly larger ON-responses in emmetropic eyes for standard contrast for the stimulus sizes 12 degrees and 6–12 degrees (Figs. 7C, 7E). Emmetropes had also larger PhNR for the 12 degrees stimulus filled with standard than with inverted contrast text (see Fig. 7C). Regarding the myopic group, their PhNR for the stimulation within a 6 degrees area were significantly larger for inverted than standard contrast (Fig. 7B).

### Prolonged Selective ON/OFF Stimulation

#### Statistical Analysis of ERG Amplitudes and Implicit Times

**Figure 8.**
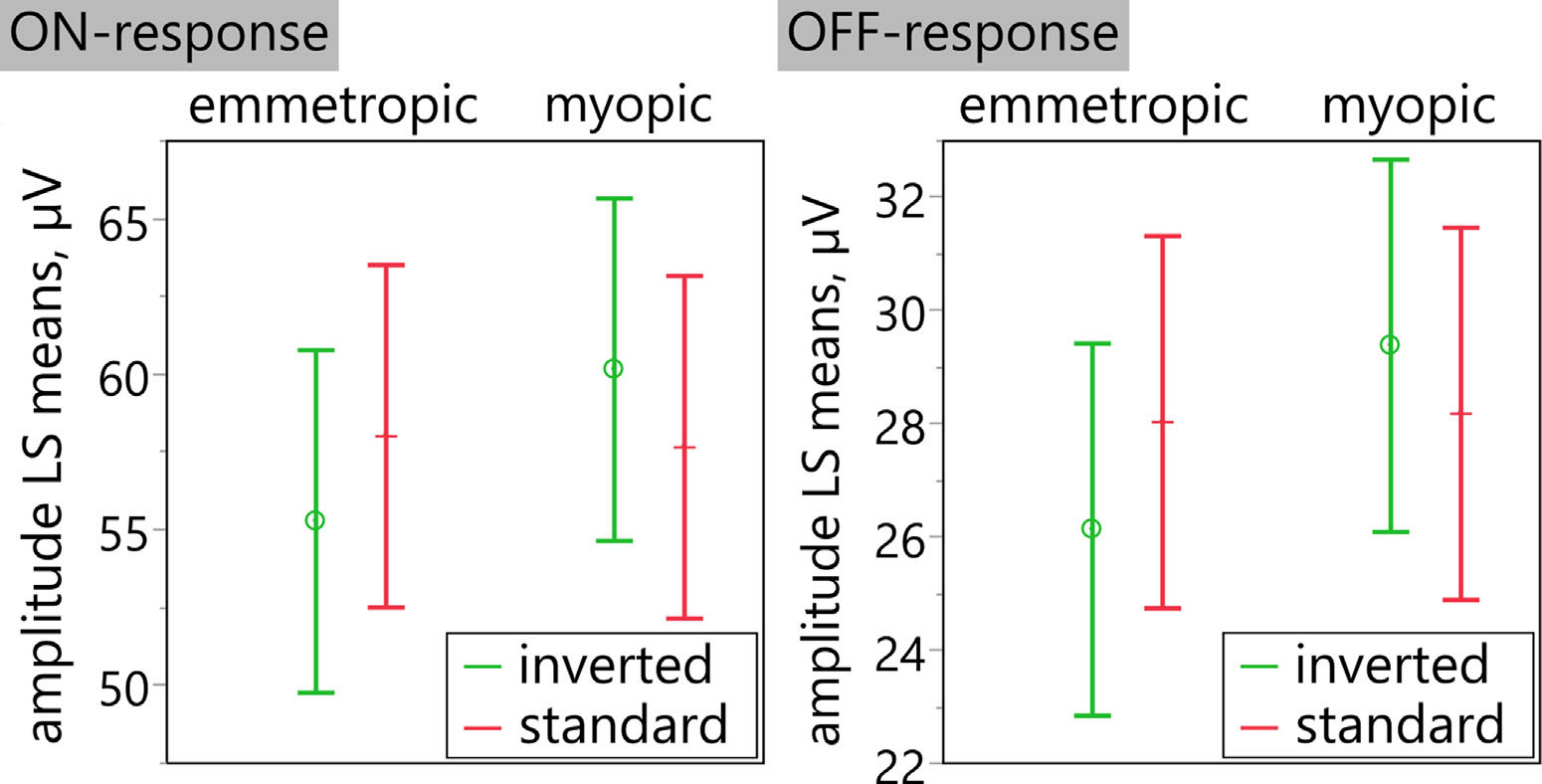
Least squares mean plots for interaction between refractive group and condition regarding retinal ON- (*left*) and OFF-responses (*right*). Error bars denote 95% *t*-type confidence limits.

The LMM analysis of the b- and d-wave amplitudes of the On-Off ERG before versus after the nearwork task revealed statistically significantly reduced ON-responses after the adaptation period, irrespective of refractive group or condition (F_1,287_ = 6.67, *p* = 0.010, Δ_pre-post_ = 1.82 µV, 95% CI = 0.43 to 3.21). There was a significant interaction of refractive group and condition (F_1,287_ = 13.77, *p* < 0.001), with significantly smaller ON-responses in emmetropic eyes for inverted than for standard contrast (Δ_st-inv_ = 2.74 µV, 95% CI = 0.16 to 5.32, *p* = 0.032; Fig. 8 left). The OFF-responses showed a significant interaction of refraction and condition (F_1,287_ = 9.85, *p* = 0.002): Emmetropes had significantly smaller OFF-responses in case of inverted than with standard contrast text (Δ_st-inv_ = 1.90 µV, 95% CI = 0.09 to 3.70, *p* = 0.035; see Fig. 8 right).

#### Comparison of Retinal Responses Before Versus After Reading Task

**Figure 9.**
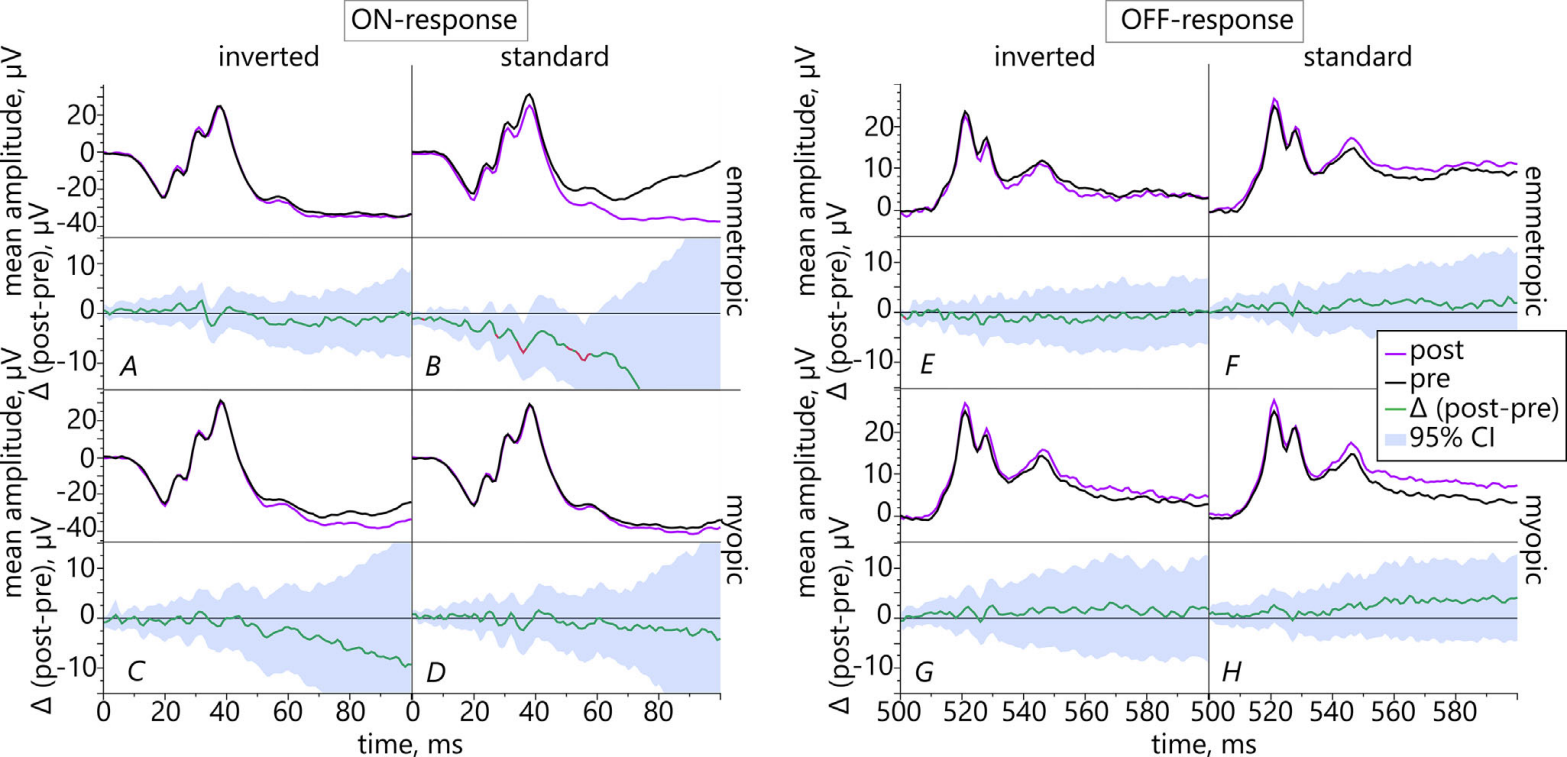
Retinal ON- (*left*) and OFF-responses (*right*) of emmetropic (*top row*, *n* = 21) and myopic subjects (*bottom row*, *n* = 21) before (*black*) versus after the reading task (*purple*) using inverted and standard contrast text. The green graphs show the mean differences Δ(post-pre) and the respective 95% confidence intervals (significant results are *highlighted in red*).

We used pointwise t-testing with 95% CIs to assess the subjects’ ERG responses over the entire recording time and compared the results before versus after the task. The analysis revealed that emmetropes had statistically significantly reduced ON-responses after the reading period with standard contrast text (Fig. 9B).

#### Comparison of Retinal Responses to Standard Versus Inverted Contrast Text

**Figure 10.**
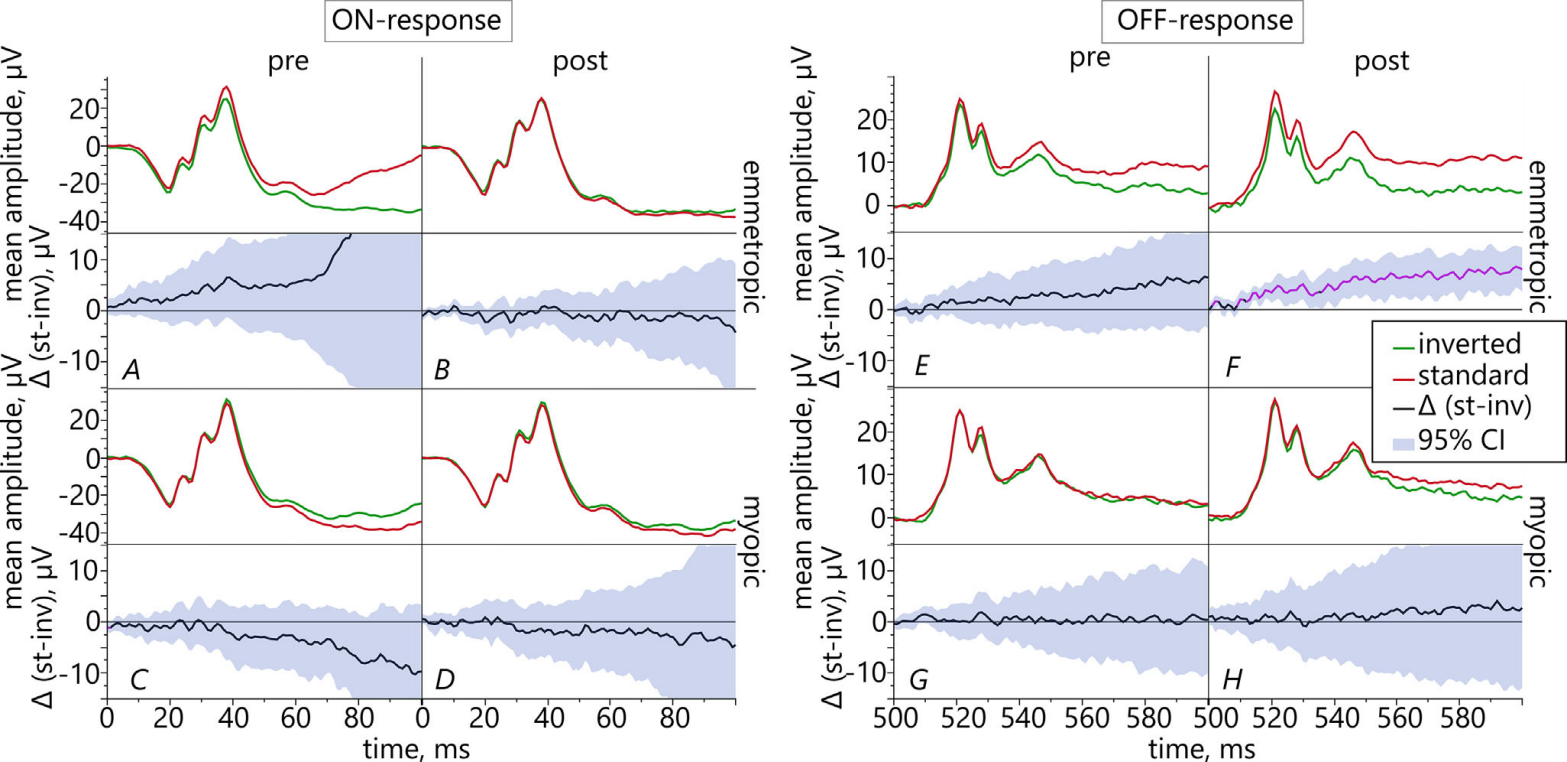
Pairwise comparison between ERG responses with different contrast polarities in emmetropic (*top row*) and myopic participants (*bottom row*) before (*left columns*) and after the reading task (*right columns*), separately illustrated for retinal ON- (*left*) and OFF-responses (*right*). Black graphs show mean difference between the 2 reading conditions Δ(standard-inverted) with 95% confidence intervals. The *pink* color marks significant result in mean differences.

The pointwise *t*-test analysis showed statistically significantly increased OFF-responses in emmetropic eyes for text of standard as compared to inverted contrast after the reading task (Fig. 10F).

#### Comparison of Retinal Responses of Emmetropic Versus Myopic Subjects

**Figure 11.**
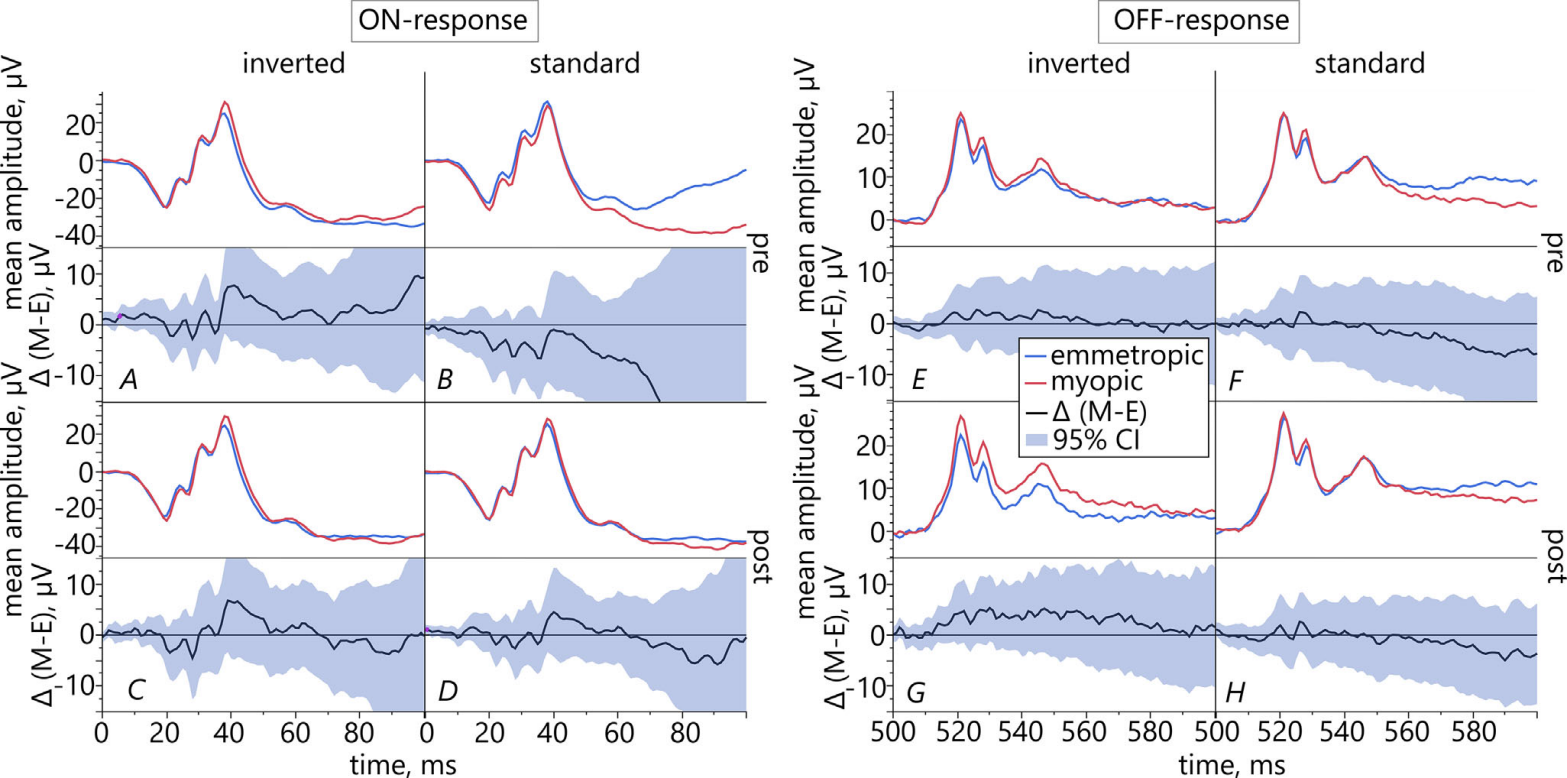
Comparison between differences of the means (*black*) of myopic (*red*) and emmetropic (*blue*) ERG responses with 95% confidence intervals, separately depicted for retinal ON- and OFF-responses.

The pairwise comparison between the two refractive groups did not reveal any statistically significant differences as for their retinal ON- and OFF-responses (Fig. 11).

## Discussion

The odds for a child to become myopic are driven by genetic and environmental factors, and nearwork was suggested to lead to an increased risk. The cause-effect relationship of reading and myopia, however, has remained unknown until today, and evidence is ambiguous.^40^ The recent finding that text contrast polarity influences the eye's axial length by activating the retinal ON or OFF channels^17^^,^^33^ initiated a new line of research. Previous studies have concentrated on anatomic ocular changes, like choroidal thickness and axial length in response to selective pathway activation.^17^^,^^29^^,^^41^ We introduced electrophysiology to fathom the effects on the retina^31^ – the origin of the ON/OFF channel division and, presumably, the controller of ocular growth. Using text contrast polarity as a means for selectively stimulating the retinal pathways, we evaluated the retinal responses to long flash ERGs both during the exposure as well as after an adaptation time of 30 minutes. We revealed (1) immediate retinal response changes during the ON/OFF stimulation: (1.1) emmetropes had higher retinal ON-responses for standard contrast text at all tested retinal areas. In myopes, the photopic negative responses were largest for uniform gray in the parafoveal area. (1.2) Myopes showed larger ON-responses to inverted contrast than emmetropes in the stimulation area of 6 degrees to 12 degrees. (2) We furthermore found significant retinal response changes after 30 minutes of adaptation to selective ON/OFF stimulation: (2.1) ON-responses were reduced with both text contrasts in both refractive groups. (2.2) Emmetropes showed reduced ON- and OFF-responses with inverted text contrast. After adapting to standard contrast text, emmetropes had reduced ON- and larger OFF-responses.

The presented differences between ERG responses of emmetropic and myopic eyes require further investigation to determine whether they explain how reading possibly triggers eye elongation.

### Immediate Retinal Changes to ON/OFF Stimulation

#### Emmetropic ON-Responses Increased for Standard Contrast Text, Whereas Myopes Showed Largest Retinal Changes to Gray

Regarding the simultaneous stimulation with either an ON, an OFF or a uniform gray stimulus, the separate group analysis revealed higher ON-responses in emmetropes at all tested retinal areas for the standard contrast text as compared to the responses to inverted contrast and uniform gray. This conflicts with a previous investigation which reported that stimulation with standard text led to higher activity in OFF than ON pathways within the foveal area.^30^ In our study, myopic eyes did not show any significant differences in their retinal ON-/OFF-responses for the three fillings of the patterns, namely standard contrast, inverted contrast, and uniform gray. Rather, myopes showed effects on their retinal ganglion cell (RGC) reactions. These were stronger for the gray stimulus than for the 2 contrast polarities for a stimulation area up to 12 degrees. Moreover, their RGC activity for foveal stimulation was higher in case of ON than OFF stimulation. Inverted contrast text provides higher spatial and temporal contrast at the fovea than standard contrast text, and this difference increases with optical blur.^30^ The increased foveal responses in myopes with white-on-black text could thus derive from optical blur due to accommodation inaccuracy. Excessively increased lags of accommodation are more often found in myopic eyes.^42^ Overall, the results of the pairwise comparisons between the groups and between the conditions provided evidence that myopes exhibit stronger retinal responses to the gray stimulus than emmetropes. Applying a modified pattern ERG, we recently found significantly higher responses to gray in myopic compared to emmetropic eyes within the foveal 6 degrees area, but similar responses in the perifoveal area of 6–12 degrees.^31^ Using the On-Off ERG now allowed us to separately assess both retinal channels. We thereby revealed higher ON-responses to uniform gray in myopes within foveal (6 degrees) and parafoveal regions (12 degrees), but not regarding the annular area of 6–12 degrees around the fovea. In contrast, the OFF-responses of myopes to gray were significantly higher than in emmetropes for parafoveal and perifoveal stimulation, but not for foveal stimulation (central 6 degrees). The question arises whether the higher foveal sensitivity to gray in myopic eyes, which was measured in the pattern ERGs, could result from a larger impact of the retinal ON pathway in the ON/OFF ratio. An imbalance between ON/OFF channels in myopic eyes has been suggested before: measuring ERGs in children with retinopathy of prematurity (ROP), Fulton & Hansen found stronger ON- than OFF-responses in myopic eyes or eyes developing myopia.^43^ The authors suggested that an imbalance of the ON/OFF ratio toward increased ON contribution might predict myopia onset in patients with ROP.^43^ However, high myopia associated with ROP might rather be related to changes in the anterior segment of the eye than being based on excessively longer eyes.^44^^,^^45^ A recent study in healthy adults with various refractive errors found that reduced ON pathway contributions are related to myopia.^46^ This is also in line with investigations of patients with complete congenital stationary night blindness (cCSNB). The inherited retinal disease is characterized by high myopic refractive errors, reduced visual acuity, and a lack of the b-wave amplitude in full-field ERG recordings.^47^^–^^49^ Furthermore, studies in the mouse model showed that animals with ON pathway mutations were more prone to develop experimentally induced myopia.^50^

The distribution of cone photoreceptors^51^ reaches its maximum at the central 0.032 degrees². This could explain the increased retinal responses in the foveal area of 6 degrees found in both groups. However, independent of refraction and polarity, we measured smaller responses in the 12 degrees region than in the perifoveal annulus of 6–12 degrees. One could argue that the measured responses reflect the retinal changes induced by the area which is illuminated during the long flash ERG rather than by the pattern filled with text or gray. If this was the case, the annulus stimulus would elicit the largest retinal response because the pattern omits the part of the retina with the largest photoreceptor density. However, this is not confirmed by our data. We therefore suggest that the implemented patterns may cause a form of inhibition or excitation, which could explain the observed differences in retinal responses to the flash stimulus.

#### Importance of Perifoveal Area for Stimulation of Myopic Retina

Another important finding was that myopic retinae showed larger ON-responses than emmetropic eyes for inverted contrast when the perifoveal area was stimulated. This retinal region was found to play a crucial role in the onset or progression of myopia, for example, it was suggested to be responsible for deciphering the sign of defocus.^52^ A recent study found that positive defocus is mainly detected in the near-peripheral retina.^53^ Others found evidence that peripheral blur orientation provides a cue for distinguishing positive from negative defocus.^54^^–^^56^ Irrespective of the refractive error, Poudel and colleagues reported a larger effect on the peripheral than the central retina after 5 minutes of reading standard contrast text.^30^ Selective activation of the perifoveal retina with a predominant ON stimulus, such as text of inverted contrast might therefore attenuate myopia progression. Increased sensitivity to this type of stimulus in myopic eyes makes the 6–12 degrees area a promising candidate for further investigating the selective ON/OFF stimulation as a means for reducing myopia progression. On the other hand, our finding of increased ON-responses to OFF stimuli in emmetropic retinae becomes important if considering the selective pathway activation as a method for preventing myopia onset.

### Adaptation Mechanisms After Reading Period With ON or OFF Channel Activation

#### No Matter Which Text Contrast, Short-Distance Reading Reduced ON-Responses in Emmetropes and Myopes

A prolonged nearwork period of ON or OFF pathway stimulation using an isoluminant text of inverted or standard contrast at short distance results in a reduction of the retinal ON-responses in emmetropic and myopic eyes. This effect might derive from the short viewing distance, which was already found to attenuate visual responses of ON more than of OFF pathways.^30^ Moreover, subjects might not have accommodated accurately during the reading phase, resulting in a lag of accommodation. A sustained under-accommodation over 30 minutes would induce the experience of defocus. It was previously reported that defocus also results in a weakening of the visual ON-responses rather than affecting the OFF-responses.^57^^–^^59^ This might be explained by the previous finding that optical blur, removing high spatial frequency content from the stimulus, changes the cortical ON/OFF balance toward a cortical OFF dominance.^57^ Our outcome of reduced ON-responses after reading with both text contrast polarities emphasizes the necessity to develop appropriate means to exclusively activate the retinal ON pathway. This is especially required in schoolchildren being at risk of developing myopia.

#### Reading Inverted Contrast Text Might at Least Reduce the OFF Channel Activation

Furthermore, we provide evidence for emmetropic eyes being more sensitive to text of different contrast polarity. We had expected that text of inverted contrast increases ON-responses. However, the retinal responses of emmetropes, both in the ON and OFF channels, were significantly reduced with inverted as compared to standard contrast text. Using the pointwise analysis confirmed that only emmetropic eyes exhibit significant changes in retinal responses after reading. They had reduced ON-responses after OFF stimulation and reduced OFF-responses after ON stimulation. Hence, whereas the stimulation with inverted contrast text was not sufficient to selectively activate the retinal ON channel, our intervention at least helped to reduce the OFF channel activation. This might already make a difference because an increased stimulation of this pathway was found to induce thinner choroids and longer eyes, and thereby possibly contribute to myopia development.^17^^,^^33^

A previous clinical study reported smaller retinal responses in the pattern ERG the longer the eye.^60^ Westall et al. found a linear reduction in the logarithmic ERG amplitude with increasing axial length, whereas the implicit times were not significantly affected.^61^ Moreover, myopia is associated with defects in the visual ON pathway.^16^^,^^17^^,^^62^ A recent study including subjects with a broad range of refractive errors measured weaker ON- and OFF-responses as well as increased implicit times for the retinal ON-responses in longer eyes.^46^ Our results, however, do not show a generally lower b-wave amplitude in myopic eyes. This could be due to lower myopic refractive errors in our cohort, ranging between spherical equivalents of –4.88 D to –0.75 D as compared to the cohort of Poudel and colleagues, reporting a maximum SER of –12.5 D.^46^ It could be that it played a role if the myopia was stable or progressive or refractive versus axial. In addition, with a maximum of 1000 cd/m², these authors had used much higher light intensities during their ERG recordings^46^ compared to 330 cd/m² in the present experiment. In contrast to our findings in emmetropic eyes, the responses in myopic eyes are not significantly affected by the close work with text of either polarity or differ between the stimulation conditions. This finding might point to changes in their retinal processing, perhaps compromising the mechanism of emmetropization.^63^^,^^64^ Given that an emmetropic (adult) eye behaves in the same way as a yet emmetropic developing eye of a child, our result suggests that selective retinal ON pathway stimulation as a means for myopia control might be more useful before myopia onset.

A Limitation of Our Methodology Is That On-Off ERGs Do Not Warrant the Clear Separation Between Retinal ON- and OFF-Responses. The simultaneous stimulation of these pathways using patterns filled with text of either contrast polarity only simulates the reading experience: Eye movements including saccades, regression, and fixation time^65^ are not present while taking the long flash ERGs. Moreover, retinal response changes in children might differ from those in young adults. It needs to be considered that myopia is multifactorial. The selective ON/OFF channel activation of a myopic eye might thus induce ERGs that possibly deviate from the results of our cohort. Further limiting factors are that the tested subjects were mostly female and of Caucasian/White background with rather light iris color, which both might have affected the outcome. As for the statistical evaluation of the ERGs, we want to emphasize that our pointwise *t*-test analysis does not consider multiple testing, nor possible correlations between adjacent time points. However, false positive results would be distributed throughout the time series with a low probability of occurring consecutively. Our approach allowed us to include all ERG data without being limited to specific features of the ERG response. Instead of peaks and troughs, our analysis used time ranges attributed to the respective ERG-wave (a, b, d, and PhNR), and thereby provided strong evidence for differences between the groups and conditions tested.

This is the first study assessing immediate and adaptation effects to selective retinal ON/OFF channel stimulation during nearwork in eyes of different refractive error. Using electrophysiology, we show that (1) retinal changes are already measurable during the separate channel activation and that these effects differ between emmetropic and myopic eyes and between central and peripheral retinal areas. This study reveals (2) that prolonged selective ON/OFF pathway stimulation during close work influences the retinal activity, however being significant in emmetropic eyes only.

Although our study is of exploratory nature, it has implications for further investigations of the retinal ON/OFF system and its relation to the refractive error. We showed that the features of the reading text affect the retinal activity. In view of the suggested link between reading and myopia, these findings might be the cornerstone for improving current myopia control strategies.

## Supplementary Material

Supplement 1
