# Supplementary material for: Retinal Responses to Short- and Longer-Term Predominant ON or OFF Stimulation in Emmetropes and Myopes
Source: Invest Ophthalmol Vis Sci. 2025 Feb 26;66(2):66. doi: 10.1167/iovs.66.2.66 (PMC11875035; doi:10.1167/iovs.66.2.66)
Supplement: Supplement 1 [file iovs-66-2-66_s001.pdf]

Supplementary material

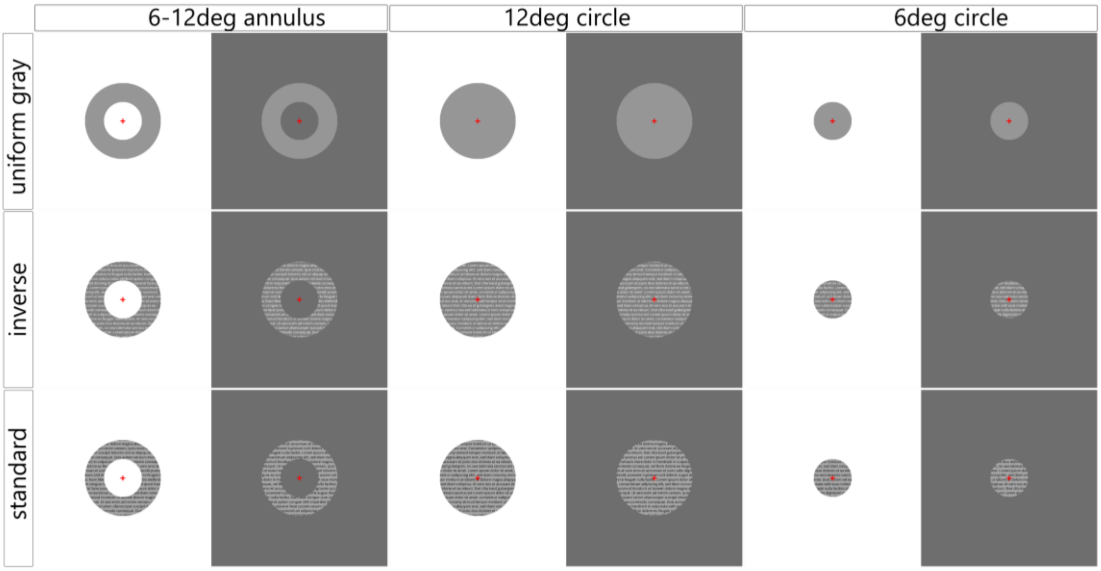

Supplementary Figure 1: Stimuli for simultaneous ON or OFF stimulation (experiment 1) using long flash ERG. Subjects were asked to fixate on the red cross during the recording.
